# Kihito, a Traditional Japanese Kampo Medicine, Improves Cognitive Function in Alzheimer's Disease Patients

**DOI:** 10.1155/2019/4086749

**Published:** 2019-05-14

**Authors:** Hidetoshi Watari, Yutaka Shimada, Mie Matsui, Chihiro Tohda

**Affiliations:** ^1^Department of Japanese Oriental Medicine, Graduate School of Medicine and Pharmaceutical Sciences, University of Toyama, 2630 Sugitani, Toyama 930-0194, Japan; ^2^Laboratory of Neuropsychology, Kanazawa University, Kakuma-machi, Kanazawa, 920-1192, Japan; ^3^Division of Neuromedical Science, Institute of Natural Medicine, University of Toyama, 2630 Sugitani, Toyama 930-0194, Japan

## Abstract

**Background and Aims:**

We previously reported that the administration of traditional Japanese medicines, kihito (Gui-Pi-Tang in Chinese) and kamikihito (Jia-Wei-Gui-Pi-Tang in Chinese), to Alzheimer's disease (AD) model mice improved memory impairment. There are a few reports that show kihito and kamikihito have a beneficial effect on the cognitive function of AD patients in clinical studies. However, these studies are not comparative and are retrospective studies; thus, more evidence is needed. Therefore, we conducted an open-label, crossover designed clinical trial to investigate the effect of kihito on cognitive function of AD patients.

**Methods:**

The inclusion criteria for eligible patients were as follows: (1) imaging diagnosis (magnetic resonance imaging and single-photon emission computed tomography) of AD, (2) a treatment regimen including acetylcholinesterase inhibitors (ChEIs), and (3) a Mini-Mental State Examination (MMSE) score ≥15. The exclusion criteria were as follows: (1) change in ChEI dosage, (2) memantine usage, and (3) MMSE score < 15. To prevent bias in age and baseline cognitive function, patients were divided into two groups: the first group received 2.5 g of kihito extract 3 times/day during the first half of the study (weeks 0-16) and the second group received the same dose of kihito during the second half of the study (weeks 17-32). ChEI dosage did not change during the study period. Patients underwent a cognitive function test during weeks 0, 16, and 32. Cognitive function was evaluated by Japanese versions of the Mini-Mental State Examination (MMSE-J) and the Repeatable Battery for the Assessment of Neuropsychological Status (RBANS-J) test.

**Results:**

Ten patients completed the clinical trial (4 males, 6 females, average age 71.7 years). MMSE-J scores significantly increased during the kihito intake period. RBANS-J test scores had a slight improvement during the kihito intake period compared with the ChEI alone treatment period, but no significant changes were observed.

**Conclusion:**

Kihito improves cognitive function in AD patients.

## 1. Introduction

Alzheimer's disease (AD) is the primary cause of dementia. Also, as the elderly population increases, the number of patients with AD is steadily increasing as well [1]. Due to the nature of the disease, cognitive decline is a major problem for AD patients. Moreover, AD imposes economic, time, and mental burden on the patient's family; these burdens have a substantial influence on society [2]. Acetylcholinesterase inhibitors (ChEIs) and N-methyl-d-aspartate (NMDA) receptor antagonists are currently the only approved drugs for AD treatment, but these drugs do not reverse the progression of disease [3]. Hence, there is an urgent necessity to establish curative treatments for AD. Many related clinical trials have been performed worldwide; in spite of numerous efforts, all approaches to improving AD prognoses such as monoclonal antibodies that bind to amyloid beta (A*β*) [4], *β*-secretase [5], and gamma (*γ*)-secretase [6] inhibitors have been unsuccessful. Consequently, we explored traditional Japanese Kampo medicine as a potentially effective option for AD treatment, focusing specifically on kihito (Gui-Pi-Tang in Chinese) and kamikihito (Jia-Wei-Gui-Pi-Tang in Chinese). Kihito, a Kampo formula consisting of 12 herbs, was used in this study. The 12 herbs in kihito include Ginseng Radix (*P. ginseng* C.A. Meyer), Polygalae Radix (*P. tenuifolia* Willd.), Astragali Radix (*A. membranaceus* Bunge), Zizyphi Fructus (*Zizyphus jujube* Mill. var.* inermis* Rehd.), Zizyphi Spinosi Semen (*Z. jujube* Mill. var.* spinosa*), Angelicae Radix (*Angelica acutiloba* Kitagawa), Glycyrrhizae Radix (*Glycyrrhiza uralensis* Fisch), Atractylodis Rhizoma (*Atractylodes japonica* Koidzumi ex Kitamura), Zingiberis Rhizoma (*Zingiber officinale* Roscoe), Poria (*Poria cocos* Wolf), Saussureae Radix (*Saussurea lappa* Clarke), and Longan Arillus (*Euphoria longana* Lam). Kamikihito contains those same herbs and two additional herbs: Bupleuri Radix (*Bupleurum falcatum* Linn.) and Gardeniae Fructus (*Gardenia jasminoides* Ellis).

In our previous study, administering kamikihito to AD model mice for 15 days improved memory impairments and reversed the degeneration of cortical axons and presynaptic terminals [7]. In addition, kihito administration for 3 days improved A*β*-induced impairments in memory acquisition, memory retention, and object recognition memory in mice and promoted axonal and dendritic extensions in A*β*-induced atrophied neurons [8].* In vitro*, kamikihito normalized hyperphosphorylated tau in A*β*-treated cortical neurons, resulting in axonal repair [9]. According to our previous results, the dephosphorylation effect of kamikihito on tau is expected to reconstruct neural circuits; therefore, these effects could be beneficial for AD patients.

Furthermore, the beneficial effects of kihito and kamikihito on the cognitive function of AD patients have been demonstrated in clinical trials. AD patients showed significant cognitive improvements after 3 months of treatment with kihito [10] and cognitive improvements after 9 months of treatment with kamikihito [11]. However, these studies are retrospective and do not include advanced cognitive function tests, so more evidence is needed to better elucidate their efficacy. Therefore, we conducted an open-label, crossover designed clinical trial to investigate the effect of kihito on the cognitive function of AD patients, evaluated with an advanced cognitive test. We used kihito extract in this study because kihito and kamikihito were similarly effective in our previous studies and the long-term intake of Gardeniae Fructus, a component of kamikihito, is suspected as a possible cause of mesenteric phlebosclerosis [12]. Also, in our previous* in vivo *study, the efficacies of kihito and kamikihito in memory improvement in AD model mice were very similar (data not shown).

## 2. Methods

### 2.1. Trial Design and Participants

This open-label, crossover study was conducted with the approval of the Ethics Committee of the University of Toyama. All patients provided written informed consent. The recruitment period was from July 2016 to March 2018. All the patients underwent brain magnetic resonance imaging (MRI) (or computed tomography if MRI was contraindicated) and single-photon emission computed tomography (SPECT) to exclude alternative causes of dementia. The inclusion criteria for eligible individuals were as follows: (1) AD diagnosis via imaging (MRI and SPECT), (2) a current treatment regimen including ChEIs, and (3) a Mini-Mental State Examination (MMSE) score ≥ 15. The exclusion criteria were as follows: (1) variations in ChEI dosage, (2) memantine use, and (3) an MMSE score < 15.

**Figure 1:**
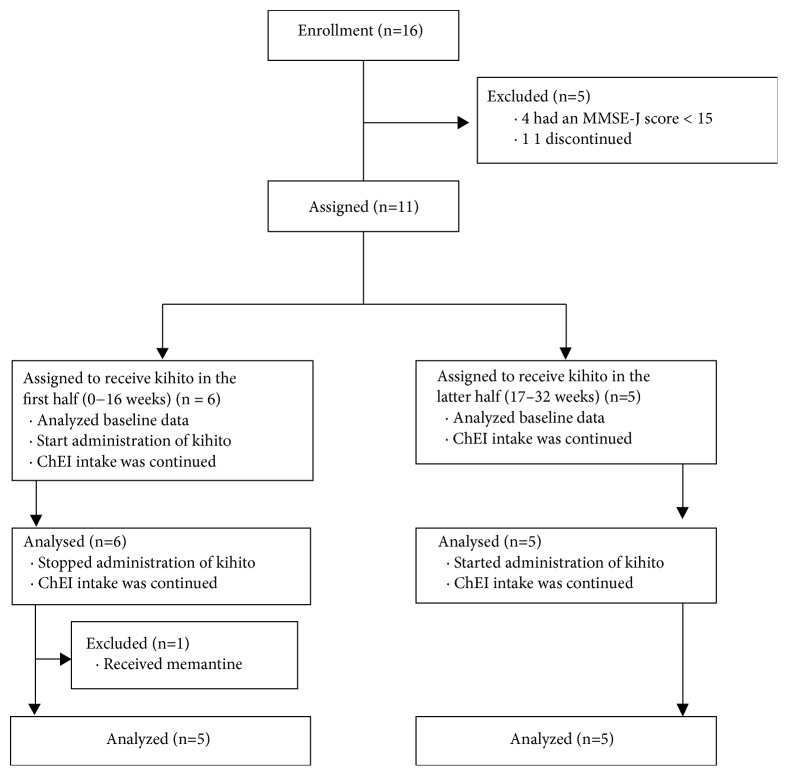
Overview of the study design. MMSE, Mini-Mental State Evaluation; ChEI, acetylcholinesterase inhibitor.

To prevent bias in age and baseline cognitive function, patients were divided into two groups: the first group received kihito extract during the first half of the study (weeks 0-16) and the second group received the same dose of kihito during the second half of the study (weeks 17-32). The ChEI dosage for each patient upon enrolment was maintained throughout the entire study. All patients visited the Toyama University hospital 3 times for assessment. An overview of trial design is shown in Figure 1.

### 2.2. Intervention

The kihito extract used in this study was purchased from Tsumura (TJ-65, Tokyo, Japan). All patients received 2.5 g of kihito extract 3 times/day during their designated times, either during weeks 0-16 or weeks 17-32. Daily doses of kihito extract include dried herbs in the following amounts: Ginseng Radix (3.0 g), Polygalae Radix (2.0 g), Astragali Radix (3.0 g), Zizyphi Fructus (2.0 g), Zizyphi Spinosi Semen (3.0 g), Angelicae Radix (2.0 g), Glycyrrhizae Radix (1.0 g), Atractylodis Rhizoma (3.0 g), Zingiberis Rhizoma (1.0 g), Poria (3.0 g), Saussureae Radix (1.0 g), and Longan Arillus (3.0 g)

### 2.3. Outcomes

Patients underwent a cognitive function test during weeks 0, 16, and 32. The primary efficacy of kihito was evaluated by Japanese versions of the MMSE (MMSE-J) and the Repeatable Battery for the Assessment of Neuropsychological Status (RBANS-J).

### 2.4. Neurocognitive Assessments

The MMSE-J score is used globally for evaluating the severity of cognitive impairment. Since the test can be carried out in a relatively short time, it is suitable for screening dementia. It consists of 11 items that refer to different cognitive domains: temporal orientation, spatial orientation, memory registration, attention and calculation, delayed memory recall, language, and visuoconstructional ability.

The RBANS-J, a representative, clinician-administered neuropsychological test for adults, was used to assess multiple cognitive function domains [13]. This test includes 12 standard cognitive subtests grouped into the following 5 domains: immediate memory (list learning and story memory), visuospatial/constructional (figure copying and line orientation), language (picture naming and semantic fluency), attention (digit span and digit symbol coding), and delayed memory (list recall, list recognition, story recall, and figure recall). The reliability and validity of the Japanese version of the RBANS have been well-established [14], and at least two forms were prepared to avoid the effect of learning via test repetition. In both MMSE-J and RBANS-J assessments, an increase in the score indicates improved cognitive function.

### 2.5. Safety Assessment

The safety assessment included recording adverse events and blood test results (complete blood cell counts and serum chemistry) to assess hypokalemia, liver and renal function, and anemia during each visit. A kihito regimen, according to the drug package insert, can lead to side effects such as pseudohyperaldosteronism, myopathy, anorexia, rash, abdominal pain, and diarrhea.

### 2.6. Statistical Analysis

The results are expressed as means with standard deviations (SDs). Statistical comparisons were performed using GraphPad Prism 5 (GraphPad Software, La Jolla, CA, USA). All data were analyzed using two-tailed paired* t*-tests, and* p* values < 0.05 were considered significant.

## 3. Results

**Figure 2:**
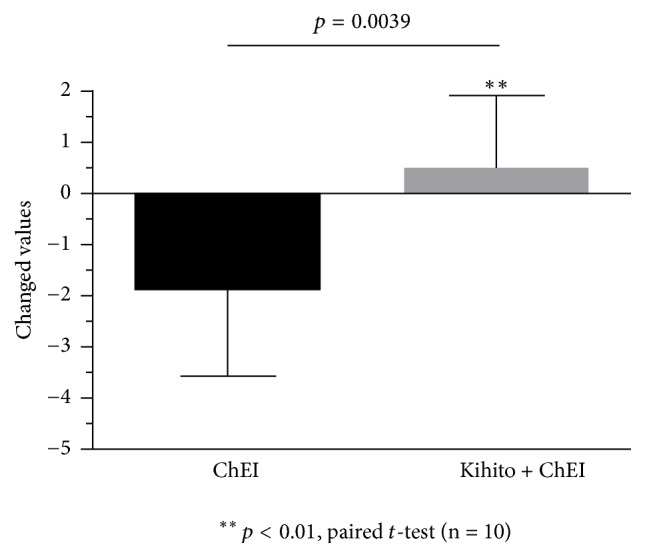
Effect of kihito extract on cognitive function in Alzheimer's disease patients based on Japanese Mini-Mental State Examination (MMSE-J) scores. Changed values of total MMSE-J scores during the acetylcholinesterase inhibitor (ChEI) alone treatment period and kihito intake period are shown. The score significantly increased during the kihito intake period compared with the ChEI alone treatment period (n = 10,* p* = 0.0039, paired t-test).

An overview of the study is shown in Figure 1. Sixteen individuals registered for this study. At the time of enrolment, 4 patients were excluded because they had MMSE scores below 15. One person chose to discontinue during the initial period. Of the remaining 11 patients, 6 received kihito during the first half of the study and 5 received kihito during the second half. However, one patient receiving kihito in the first half of the study was prescribed memantine during the trial and was subsequently excluded; thus, 5 people in each group completed the study schedule (Figure 1). The baseline characteristics of all the 10 patients who completed the study are shown in Table 1. The average age was 71.8 years, and the mean MMSE-J score was 20.5 (Table 1). Changed values in MMSE-J are shown in Figure 2. An MMSE-J score reduction of about 1.8 points was observed during the ChEI alone treatment period, whereas the score slightly increased during the kihito intake period, demonstrating cognitive improvement; this change was statistically significant.

**Figure 3:**
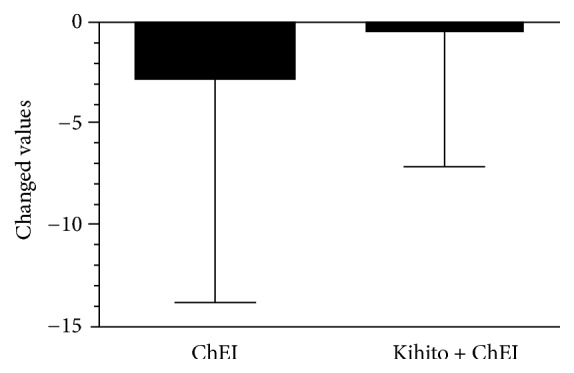
Effect of kihito extract on cognitive function in Alzheimer's disease patients based on Japanese Repeatable Battery for the Assessment of Neuropsychological Status (RBANS-J) test scores. Changed values of total RBANS-J scores during the acetylcholinesterase inhibitor (ChEI) alone treatment period and kihito intake period are shown. There were no significant changes (n = 10,* p* = 0.6268, paired t-test).

Changed values in RBANS-J are shown in Figure 3. During the ChEI alone treatment period, scores decreased by about 2.7 points, whereas during the kihito intake period there was less of a decline although the change was not statistically significant.  Table 2 displays these score changes and includes the subtest analysis of RBANS-J scores. The RBANS-J subtest results revealed that values increased in the language domain, but the change was not significant. However, there was a significant increase of 2.3 points in the average of the total MMSE-J score between the kihito intake period and the ChEI alone treatment period (Table 2). The results of the subtest analysis of the MMSE-J scores are shown in Table 3. Significant cognitive improvements were recognized in the temporal orientation test, but there were no significant changes in the other subtests (Table 3). No side effects or adverse events were observed during safety assessment visits.

## 4. Discussion

This study showed that kihito is useful for improving cognitive function in AD patients. In previous clinical studies, MMSE scores of AD patients (n = 20) improved significantly after taking 7.5 g/day kihito extract for 3 months [10]. Additionally, combination therapy consisting of 7.5 g/day of kamikihito and donepezil (n = 6) for 9 months improved the MMSE score of AD patients compared to the administration of donepezil alone (n = 6) [11]. Both results are similar to our study findings. Our study provides additional evidence regarding the benefits of kihito, especially for AD patients. Meanwhile, previous MMSE subtest analyses revealed that the numerical value of attention was improved [10] as was delayed memory [11]. These results are different from our study findings. Therefore, kihito might not affect a specific cognitive domain. Although the administration periods in the other studies are different from our research, the duration of administration is unlikely to cause specific cognitive domains to be affected differently. To elucidate the details of sensitive domains of cognition affected by kihito, more studies are needed with larger numbers of patients.

In kihito research regarding AD patients, no studies have used advanced cognitive function tests such as the Alzheimer's Disease Assessment Scale-cognitive subscale (ADAS-cog). However, about 1 hour is needed to complete the entire ADAS-cog test, so it is a burden to patients with severe AD. Accordingly, we used the RBANS-J assessment as an advanced cognitive function test because RBANS-J is a well-established and reliable test that can be completed within 30 minutes. As the RBANS-J assessment has up to 12 questions, its use leads to more data being collected than with the MMSE. Therefore, it is possible to evaluate more refined cognitive functions with the RBANS-J assessment.

RBANS-J scores revealed a smaller decline in cognitive function during the kihito oral administration period, but there was no significant difference compared with the ChEI alone treatment period. Individuals in this study had an average MMSE score of 20.5 at the start of the study; higher scores were reported in unsuccessful clinical trials for mild AD [4]. In other words, patients in our study had a more severe disease condition.

Repeated failures to develop therapeutic drugs indicate the need for early treatment intervention [15]. However, it is important to note that patients taking kihito showed some improvement, based on MMSE-J scores, under the conditions of present study. In addition, a study using another Kampo medicine (ninjin'yoeito) showed its effects during long-term treatment; cognitive improvements were observed 12 months after commencing the medication regimen [16]. Consequently, Kampo medicines may require long-term treatment regimens. Regarding the investigation of the effect of kihito in AD patients, further research is needed with a larger number of cases and should involve long-term tests targeting early stage AD.

Kihito effectively improved cognitive function, but the mechanisms of this effect are still unknown. Based on the results of our previous studies* in vitro*, kihito may improve the pathology of AD via calcium (Ca^2+^) entry inhibition or tau dephosphorylation. In our previous study, kihito inhibited A*β*-induced Ca^2+^ influx in cultured cortical neurons [8]. In addition, kamikihito treatment led to tau dephosphorylation in primary cultured cortical neurons that were phosphorylated by A*β* treatment [9]. Although these effects may explain a part of the mechanisms involved in kihito medicine, these phenomena were not investigated in this study.

Since kihito has many components, it is difficult to completely identify all of its mechanisms. However, each constitutive herb shows favorable possibilities. For instance, Polygalae Radix extract, a component of kihito and kamikihito, prevented memory deficits in a mouse model of AD and inhibited axonal growth cone collapse in cultured neurons that was induced by A*β* [17]. Tenuifolin, a secondary saponin from hydrolysates of polygalasaponins (active constituents of Polygalae Radix), exhibited neuroprotective effects against A*β*-induced apoptosis in PC12 cells, which are derived from the pheochromocytoma of the rat adrenal medulla, and significantly improved the cognitive deficits induced by the intrahippocampal injection of A*β* in mice [18]. BT-11, the extract of dried roots of Polygala tenuifolia, showed a memory-enhancing effect in healthy human adults [19]. These results indicate that Polygalae Radix is a key component of the beneficial effects of kihito. Notably, the synergistic and additive effect of multiple compounds is important for Kampo medicine. Examining the exquisite combinations of Kampo medicine is a critical task for future research.

We adopted a crossover study design that was advantageous in previous clinical studies with a small number of cases and was suitable for this study. However, we did not provide a washout period, which is usually used in a crossover design clinical study, to avoid a learning effect caused by repeatedly performing cognitive function tests in a short-term washout period. Since our study does not include a washout period, there is a possibility that ChEI alone treatment period has been started from a higher score in the group receiving kihito in the first half of the study. However, since the effect of kihito use was to maintain cognitive function, the possibility would not be an issue.

Several limitations in the present study should be noted. Our study had a small sample size and was not a randomized, placebo-controlled, double-blind study. Also, our treatment was studied for a relatively short-term period. Further research needs to address these limitations and to provide additional robust evidence showing the benefits of kihito for AD treatment.

## 5. Conclusion

In this prospective clinical study using a crossover design, kihito administration improved cognitive function in AD patients. It is a new finding that not only MMSE-J but also the advanced cognitive function test RBANS-J showed improvement trends with kihito use. Although further studies are needed to provide clinical evidence and to clarify the underlying mechanisms of kihito, our findings indicate that kihito use may be beneficial in dementia treatment regimens.

## Figures and Tables

**Table 1:** Baseline characteristic of the sample.

Values	
Subject (Men/Women)	10 (4/6)
Age (years, mean ± SD)	71.8 ± 6.93 (58-82)
Race	Asian
MMSE-J (mean ± SD)	20.5 ± 3.74
RBANS-J total (mean ± SD)	54.9 ± 22.2

SD: standard deviation.

**Table 2:** Changes in Japanese Mini-Mental State Examination (MMSE-J) and Repeatable Battery for the Assessment of Neuropsychological Status (RBANS-J) scores between acetylcholinesterase inhibitor (ChEI) alone treatment period and kihito intake period.

Cognitive Domain	ChEI alone		ChEI + kihito		Mean Difference	SD Difference	95% CI	*p* Value
	Baseline	16 weeks	Baseline	16 weeks				
MMSE-J	21 ± 4.42	19.2 ± 4.59	19.7 ± 4.24	20.2 ± 4.96	2.3	1.888	0.9491 to 3.651	0.0039*∗*

RBANS								
Total score	54.8 ± 26.0	52.1 ± 25.7	54.6 ± 25.3	54.3 ± 27.8	2.4	15.07	-8.386 to 13.19	0.6268
Immediate memory	61.7 ± 12.3	63.4 ± 15.4	67.8 ± 18.0	62.5 ± 13.4	-7.0	20.40	-7.609 to 21.61	0.3066
Visuospatial/Constructional	83.9 ± 34.5	80.3 ± 40.4	79.4 ± 39.1	74.9 ± 40.3	-0.9	24.98	-16.97 to 18.77	0.9000
Language	75.4 ± 23.1	66.1 ± 22.2	72.1 ± 20.5	77.4 ± 25.0	14.6	23.05	-1.892 to 31.09	0.0763
Attention	76.4 ± 19.1	75.7 ± 16.1	76.8 ± 17.3	76.5 ± 21.3	0.4	13.22	-9.061 to 9.861	0.9259
Delayed memory	50.7 ± 15.4	54.7 ± 12.4	52.0 ± 13.8	52.9 ± 14.1	-3.1	21.45	-18.45 to 12.25	0.6586

SD: standard deviation; CI: confidence interval.

*∗ p *< 0.05, two-tailed paired t-test.

**Table 3:** Changes in Japanese Mini-Mental State Examination (MMSE-J) subtest scores between acetylcholinesterase inhibitor (ChEI) alone treatment period and kihito intake period.

Cognitive Domain	ChEI alone		ChEI + kihito		Mean Difference	SD Difference	95% CI	*p* Value
	Baseline	16 weeks	Baseline	16 weeks				
Temporal orientation	2.8 ± 1.87	2.1 ± 1.44	2.0 ± 1.49	2.8 ± 1.39	1.5	1.715	0.2726 to 2.727	0.0220*∗*
Spatial orientation	3.3 ± 0.94	2.8 ± 1.31	3.0 ± 1.33	3.2 ± 1.13	0.7	1.337	-0.2567 to 1.657	0.1323
Memory registration	3.0 ± 0	2.9 ± 0.31	2.9 ± 0.31	2.9 ± 0.31	0.1	0.316	-0.1262 to 0.3262	0.3434
Attention	2.4 ± 1.64	2.0 ± 1.94	2.5 ± 1.77	2.2 ± 1.81	0.1	2.282	-1.533 to 1.733	0.8929
Delayed memory recall	1.3 ± 1.15	1.3 ± 1.05	1.2 ± 0.91	1.0 ± 1.05	-0.2	1.229	-1.079 to 0.6793	0.6193
Naming	2.0 ± 0	2.0 ± 0	2.0 ± 0	2.0 ± 0	no change			
Phrase repetition	0.8 ± 0.42	0.8 ± 0.42	0.7 ± 0.48	0.7 ± 0.48	0.0	0.666	-0.4769 to 0.4769	1.00
Comprehension	2.9 ± 0.31	3.0 ± 0	3.0 ± 0	3.0 ± 0	-0.1	0.316	-0.3262 to 0.1262	0.3434
Reading	0.9 ± 0.31	1.0 ± 0	1.0 ± 0	0.9 ± 0.31	-0.2	0.632	-0.6524 to 0.2524	0.3434
Writing	0.9 ± 0.31	0.7 ± 0.48	0.7 ± 0.48	0.8 ± 0.42	0.3	0.483	-0.04553 to 0.6455	0.0811
Visuo‐constructional ability	0.7 ± 0.48	0.6 ± 0.51	0.7 ± 0.48	0.7 ± 0.48	0.1	0.737	-0.4278 to 0.6278	0.6783

SD: standard deviation; CI: confidence interval.

*∗ p *< 0.05, two-tailed paired t-test.

## Data Availability

The data used to support the findings of this study are included within the article.
